# The effect of heel elevation on the stiffness gradient index of the digital flexor tendons in the equine forelimb of clinically normal horses

**DOI:** 10.3389/fvets.2025.1610788

**Published:** 2025-10-01

**Authors:** Kelly A. Shaw, Sabrina H. Brounts

**Affiliations:** Department of Surgical Sciences, School of Veterinary Medicine, University of Wisconsin, Madison, CA, United States

**Keywords:** horse, equine, heel wedge, heel elevation, tendon strain, flexor tendons, acoustoelastography, shoeing

## Abstract

**Objective:**

To evaluate the effect of heel elevation on *in vivo* measurement of stiffness gradients by means of acoustoelastography in the digital flexor tendons of clinically normal horses.

**Animals:**

15 clinically normal horses.

**Procedures:**

For each horse, stiffness gradient index (SGI) for superficial digital flexor tendons (SDFT) and deep digital flexor tendons (DDFT) were evaluated in both forelimbs at 0, 4 and 8 degrees of heel elevation. Acoustoelastography (AEG) was used for data acquisition at three sites, approximately 6, 12, and 18 cm distal to the accessory carpal bone in the metacarpal region. Lifting the contralateral limb during image acquisition resulted in the application of load and the subsequent SDFT and DDFT deformation required. The effects of loaded versus unloaded digital flexor tendons and right-to-left limb symmetry on SGI at three regions in the metacarpal region were further evaluated. Changes in angulation of the metacarpophalangeal, proximal interphalangeal, and distal interphalangeal (DIP) joints in conjunction with the palmar angle (PA) of the distal phalanx and toe angle (TA) were measured radiographically at 0, 4 and 8 degrees of heel elevation to approximate trends in digital angles with applied heel elevation.

**Results:**

SGI values for SDFTs and DDFTs differed significantly in loaded versus unloaded tendons and at different locations of the metacarpus. Incremental heel elevation had the greatest and most consistent effect on the SGI in the mid-metacarpal region in both flexor tendons. At this level, the stiffness gradient tended to decrease when the angle of heel elevation increased from 0 to 8 degrees for both flexor tendons. There was a significant difference in SGI between loading and unloading the limb during AEG acquisition, with reduced variability when the digital flexor tendons were loaded.

**Conclusions and clinical relevance:**

Results indicated that the SGI of digital flexor tendons was significantly affected by heel elevation. Regarding technique, AEG can be easily and effectively utilized to measure flexor tendon strain in a standing horse with applied heel elevation. This model resulted in direct quantification of tendon strain as it relates to distal limb conformation, which would allow for more targeted therapeutic farriery techniques.

## 1 Introduction

The equine foot plays a vital role in the kinematics of locomotion, with a complex anatomy specialized for bearing and transmitting load (1). A delicate balance exists between structures providing suspension versus support in the equine distal limb, and maintaining appropriate conformation of these structures is critical for performance, duration of athletic career, and overall soundness of the horse (2). Corrective farriery is commonly prescribed in clinical cases for treatment and prevention of orthopedic disease through the influence it has on limb biomechanics. This practice utilizes particular techniques to alter the digital joint angles and affect the course of tendons relative to other anatomical structures, which have specific conformational and load-based effects with varying therapeutic implications (3–5).

Specialized shoes or orthotics have been designed to alter the bony alignment of the distal limb frequently by adjusting the palmar/plantar angle of the distal phalanx (P3) within the hoof capsule through either heel or, less frequently, toe elevation. These changes directly affect digital joint angles which, in turn, influence the strain experienced by the SDFT and DDFT by adjusting anatomical insertion sites (6). The use of orthotics has been specifically advocated for treatment of navicular disease and laminitis considering the effects exerted on the DDFT, as well as for various pathologies related directly to the flexor tendons (i.e., tendonitis, lacerations, tendon rupture) or distal phalanx (7).

Despite the widespread use of orthotics, there is limited data available which can directly quantify the effects of altering hoof conformation on the resultant strains of the digital flexor tendons in live, weight bearing horses. There are furthermore complex reciprocal interactions between the anatomical structures of the distal limb, which would make a quantitative understanding of the interrelationship between the changes in palmar angle, phalangeal joint angles, and flexor tendon strain extremely valuable for clinical decision making (8).

Tendon strain, which is defined as the increase in length of a tendon when a load is applied during muscle contraction, is calculated as the change in length divided by the initial length, and is directly associated with tendon stiffness or resistance to deformation (9, 10). Tendon stiffness is assumed to increase when the associated muscle contracts, to prevent the tendon from stretching and counteracting contraction of the muscle. A stretched tendon under increased strain has a greater ability to resist deformation, has an increased level of stiffness, and a subsequently elevated stiffness gradient index (SGI). Considering the elastic nature of tendons, SGI should vary systematically as a function of strain by decreasing when strain is reduced and increasing as strain is elevated.

While joint angles can be easily calculated from radiographic images, measurement of tendon strain *in vivo* is challenging due to the invasive nature of most strain gage devices utilized in equine tendon strain research (11, 12). As a result, alternative approaches to estimating tendon strain have been employed, such as 3D modeling, extrapolation from kinematic data, or measuring strain in cadaver limbs (6, 13, 14). To date, no quantitative method has been used *in vivo* to measure the effects of hoof angulation on tendon strain, and to relate the changes in strain to changes in joint conformation in a weight bearing animal.

This study uses a novel approach to calculate flexor tendon strain as a function of hoof biomechanics, with utilization of acoustoelastography (AEG). AEG is a post-processing ultrasound based tissue evaluation technique that estimates strain as a function of the change in echogenicity of a tissue as it undergoes deformation following the application of load, and then relates those changes to the mechanical property (i.e., strain and stiffness) of the tissue (15). This technique has been proven to be effective and reproducible in live, standing horses (15), has detected changes to the musculoskeletal unit as a result of sedation-induced relaxation (16), and has been used to detect pathology of the SDFT (17). Use of AEG in this study allowed for a non-invasive means of measuring the stiffness gradient of digital flexor tendons *in vivo*, both prior to and following application of various levels of heel elevation in clinically normal horses.

The purpose of this study was to evaluate the effects of heel elevation on *in vivo* measurements of SGI in the digital flexor tendons of clinically normal horses. Our hypothesis was that an increase in heel elevation from 0 to 8 degrees would be associated with an attenuation of the strain of the DDFT, and in both an attenuation and amplification of strain of the SDFT. Considering the distal insertion site of the DDFT, the attenuation of strain and subsequent effect on SGI is proposed to be linearly related to a progressive increase in heel elevation.

## 2 Materials and methods

### 2.1 Horses

Fifteen clinically normal horses were included in this study. Ten horses were selected from the University of Wisconsin teaching herd; the remaining five were recruited with informed owner consent from a population of client-owned animals. Horses were defined as clinically normal on the basis of owner history (no reported lameness or musculoskeletal disease) and results of an orthopedic examination. Lack of tendon pathology was additionally confirmed during preliminary ultrasonographic examination. All animal protocols were approved by the University of Wisconsin School of Veterinary Medicine Institutional Animal Care and Use Committee.

### 2.2 Experimental procedures

Hair was clipped on the palmar aspect of both thoracic limbs of each horse from the proximal aspect of the metacarpus to the level of the metacarpophalangeal joint. The skin was washed with chlorhexidine scrub followed with water to remove any dirt in preparation for an ultrasonographic examination. Each horse was sedated with a combination of detomidine hydrochloride (0.01 mg/kg, IV) and butorphanol tartrate (0.01 mg/kg, IV), and with redosing to achieve a comparable light plane of sedation, administered as needed.

Longitudinal and transverse images of the flexor tendons of the left and right thoracic limb were obtained at the proximal, mid and distal metacarpus, which measured approximately 6, 12, and 18-cm distal to the accessory carpal bone (ACB). A flexible measuring tape was adhered at the level of the ACB of each evaluated limb to ensure accurate and repeatable measurements. Images were acquired using a linear transducer on a portable ultrasound machine (GE, LogiQ E10), with a standard frequency setting of 12 MHz. Focal zone position was set at the level of both the SDFT and DDFT, and gain was subjectively maintained between 56 and 62% to optimize the image for each acquisition.

Provided no B-mode ultrasonographic abnormalities were evident in either flexor tendon on the survey scan, 3 cineloop video recordings were obtained in longitudinal orientation as the limb was loaded by shifting the horse from a baseline square stance to an increased weight-bearing stance, and then back to a baseline square stance. This was achieved by lifting the contralateral thoracic limb with flexion of the carpus such that additional weight was shifted onto the limb being scanned, and then replacing the contralateral forelimb back down to the ground. This motion was performed as fluently as possible. For all horses, the left forelimb was scanned before the right forelimb. Baseline images and cineloop videos (zero degree) were acquired first, and then the process repeated for each horse at four and eight degrees of heel elevation. A slip-on orthotic was constructed using a 4.75″ cuff (Nanric, Dalric cuff) riveted to stack of wedge pads to produce the desired level of heel elevation.

During ultrasonography, the quality of cineloop videos was ranked using an ordinal scale from 0 to 3, where 0 represented the poorest quality data (e.g., caused by the horse resisting to offload the tendons being examined, and/or technical difficulties experienced during data collection such as loss of contact) and 3 represented the highest quality data (e.g., when the horse offloaded the tendons smoothly, and/or technical difficulties were not experienced).

Subsequently, radiographic examination of the distal forelimbs was performed in 14 of the subjects used in this study. These horses were radiographed at 0, 4 and 8 degrees of heel elevation. A portable computed radiography unit (DÜRR NDT, ScanX) was used. All radiographs were generated by following a standard set-up (60 kV, 0.2 mAs/s and focus-film distance of 75-cm). For each forelimb, three lateromedial radiographs of the foot were obtained. The first radiograph focused the radiation beam over the distal interphalangeal (DIP) joint, the second over the metacarpophalangeal (MCP) joint, and the third over the MCP joint with the limb loaded (contralateral limb lifted and flexed, as performed during acquisition of the ultrasound cineloop videos).

During the procedure the horses were standing square, with all limbs bearing weight, and with both forelimbs positioned parallel on 6-cm tall wood blocks with embedded reference marks for subsequent analysis. All radiographs were monitored to check for orthopedic abnormalities for each subject, consistency of the image quality and projection. Radiographic data was collected to record the changes in joint angles in examined horses with applied heel elevation. Radiographic measurements of the digital angles were later performed for each joint (DIP, PIP, and MCP), for the palmar angle (PA) of the digital phalanx (P3) and toe angle (TA), at each level of heel elevation (0, 4 and 8 degrees) and in a loaded and unloaded position, using digital software. Preliminary data analysis noting trends of digital angles was evaluated in this study, and further analysis examining the digital angle measurements in relation to the SDFT and DDFT strain data acquired from AEG will be conducted in a subsequent study.

### 2.3 Data analysis

Uncompressed audio video interleave format files for each of the cineloop video recordings were used for SGI analyses, which were performed by use of analysis software by 1 investigator (KAS). A region of interest consistent in size and location, that was approximately the central half of the tendon section, was selected in each cineloop. Separate selections were made for the SDFT and DDFT. To maintain the same region of interest throughout the cineloop video, a region-based optical flow tracking technique was used to estimate the movement of the granular tendon pattern, or speckles, between consecutive frames (16, 18). The changes in relative pixel intensity and location over time were analyzed via AEG for each pixel. The stiffness gradients for each pixel within a 50 × 300- pixel area located centrally within the original region of interest were averaged. Estimates of the SGI were then calculated by linear regression analysis as the line of best fit (1st order function) to the stiffness-strain curve.

### 2.4 Statistical analysis

**Figure 1:**
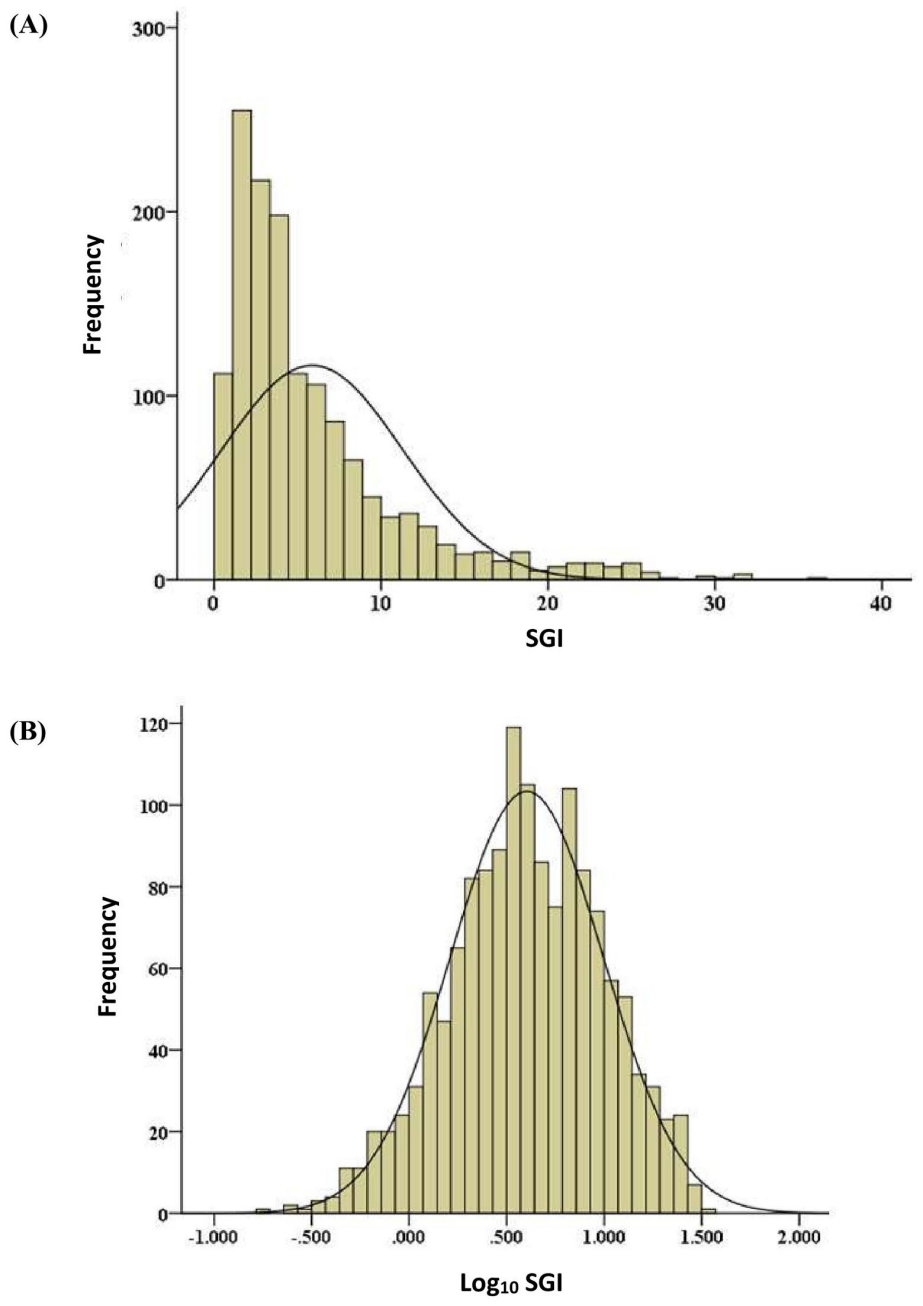
Frequency distribution of raw **(A)** and normalized log 10 transformed **(B)** SGI measurements.

All statistical tests selected and performed by a single statistician using statistical software. The data was screened and cleaned to correct measurement errors and to minimize the effects of abnormal cases on the results of inferential statistical analysis (19). Data given a subjective quality ranking >1.5 was included in the data analysis. A General Linear Mixed Model (GLMM) was constructed for analysis of repeated measures in the AEG data, as cine loops were repeated systematically for each measurement of SGI. AEG data being screened and analyzed included SGI values of the superficial and deep digital flexor tendons in both forelimbs, at 0, 4, and 8 degrees of heel elevation, at three levels in the metacarpal region (approximately 6, 12, and 18-cm distal to the ACB), and in all horses evaluated meeting the previously established inclusion criteria. Univariate outliers were identified as cases with Z-scores outside the expected normal limits of four standard deviations on either side of the overall mean SGI (20). Multivariate outliers were identified by Mahalanobis D^2^, referring to the distance of each value of SGI from the centroid in multivariate space, where the mean values of SGI with respect to each factor in the covariance matrix intersect with each other. The larger the value of D^2^, the further away was the case from the centroid (21). Outliers with Mahalanobis D^2^ values exceeding the 95^th^ percentile were ranked as poor-quality data. The total number of measurements of SGI used in the statistical analysis, excluding poor quality data and outliers totaled 1,427 (1,454 minus 27). The distribution of cleaned data (SGI data excluding outliers and poor-quality data) was evaluated and normalized with log10 transformation (Figure 1).

Effect sizes and uncertainty metrics for this data were determined. The 95% CI of the point estimates of both the unstandardized GLMM regression coefficients measured in units of log10 SGI (ß) and of the proportion of the variance in the SGI explained by GLMM (R (2)) estimated the level of uncertainty. An effect size was assumed to exhibit practical significance if the 95% CI did not capture zero; however, the 95% CI capturing zero did not imply absolutely no effect, but rather equivalent to not “statistically significant” at the 0.05 level. Considering effect size (R^2^ = 0.10, 95% CI = 0.07, 0.13), in 95 out of 100 cases about 7 to 13% of the variance in SGI was explained. The lower and upper limits of the 95% CI were both greater than 4%, indicating that the minimum effect size representing a practically significant effect was reached. 195.

## 3 Results

Of the 15 horses included in the study, 11 were mares and 4 were geldings. Horses ranged from 3 to 22 years of age (mean ± SD, 14 ± 5.4 years). Breeds of horses included Quarter Horse (*n* = 6), Thoroughbred (*n* = 2), Paint (*n* = 1), Arabian (*n* = 1), Oldenberg (*n* = 1), Missouri Fox Trotter (*n* = 1), Tennessee Walker (*n* = 1), Saddlebred (*n* = 1), and POA (*n* = 1).

The GLMM predicts the fixed effects of the examined factors (i.e., data quality, flexor tendon type, forelimb side, metacarpal region, limb load distribution, and levels of heel elevation) on the logarithmically transformed SGI measurements of cleaned data (Table 1). The point estimates of the 5 ß coefficient represented the effects of the predictor category in relation to the reference category, if there was a resultant increase (+) or decrease (−) in the SGI, assuming all other categories in the model were constant. When the 95% CI of the point estimate of the ß coefficient captured zero then the effect of the categorical factor was uncertain due to the level of variance in the SGI data (illustrated by the wide CI in Table 1).

**Table 1:** General linear mixed model to predict fixed effects of six categorical factors on Logt SGI (*n* = 1,426).

Categorical factors		Statistics				
Predictor category	Reference category	ß Coefficient	*t*	*p*	95% CI	
					Lower	Upper
Quality = 2	Quality = 3	+0.030	1.51	0.130	−0.009	+0.070
SDFT	DDFT	+0.004	0.19	0.846	−0.034	+0.042
Left limb	Right limb	−0.037	−1.84	0.065	−0.076	+0.002
Limb unloaded	Limb loaded	−0.072	−2.93	0.004	−0.121	−0.024
Proximal metacarpal region	Distal region	+0.014	0.58	0.561	−0.033	+0.061
Middle metacarpal region	Distal region	+0.030	1.24	0.215	−0.018	+0.078
Heel Elevation = 0^o^	Heel elevation = 8^o^	+0.051	2.15	0.031	+0.004	+0.098
Heel Elevation = 4^o^	Heel elevation = 8^o^	+0.016	0.69	0.493	−0.030	+0.063

Considering these principles, the GLMM provided insufficient evidence to determine if differences in the data quality (GLMM, *p* = 0.130), tendon type (GLMM, *p* = 0.846), the left versus right forelimb (GLMM, *p* = 0.065), or metacarpal region (GLMM, *p* = 0.215, 0.561) had a consistent positive or a negative effect on the SGI. With regards to the remaining categorical variables evaluated, both the degree of heel elevation and the application of load on the limb did show a more certain effect on SGI. There was a significant difference in SGI when heel elevation increased from 0 to 8 degrees in both digital flexor tendons (*p* = 0.031). There was a significant difference in SGI between loading and unloading the evaluated limb (GLMM, *p* = 0.004), where mean SGI was higher and less variable in the loaded digital flexor tendons.

Upon closer examination of the effects of heel elevation on SGI, several differences between the specific levels of heel elevation were identified. When the heel elevation was 0 degrees, the value of the point estimate of ß was +0.051 higher than when the heel elevation was 8 degrees. The 95% CI (+0.0.004, + 0.098) were consistently positive, implying that in 95 out of 100 cases, the heel elevation change from 8 to 0 degrees resulted in a concomitant increase in SGI. When the heel elevation was 4 degrees, the value of the point estimate of ß was +0.016 higher than when the heel elevation was 8 degrees and the 95% CI (−0.030, + 0.064) captured zero. Therefore, the effect of adjusting the heel elevation from 8 to 4 degrees was considerably more variable. The negative and positive CI indicated that in 95 out of 100 cases, the heel elevation change from 8 to 4 degrees did not axiomatically result in a consistent increase in SGI.

**Figure 2:**
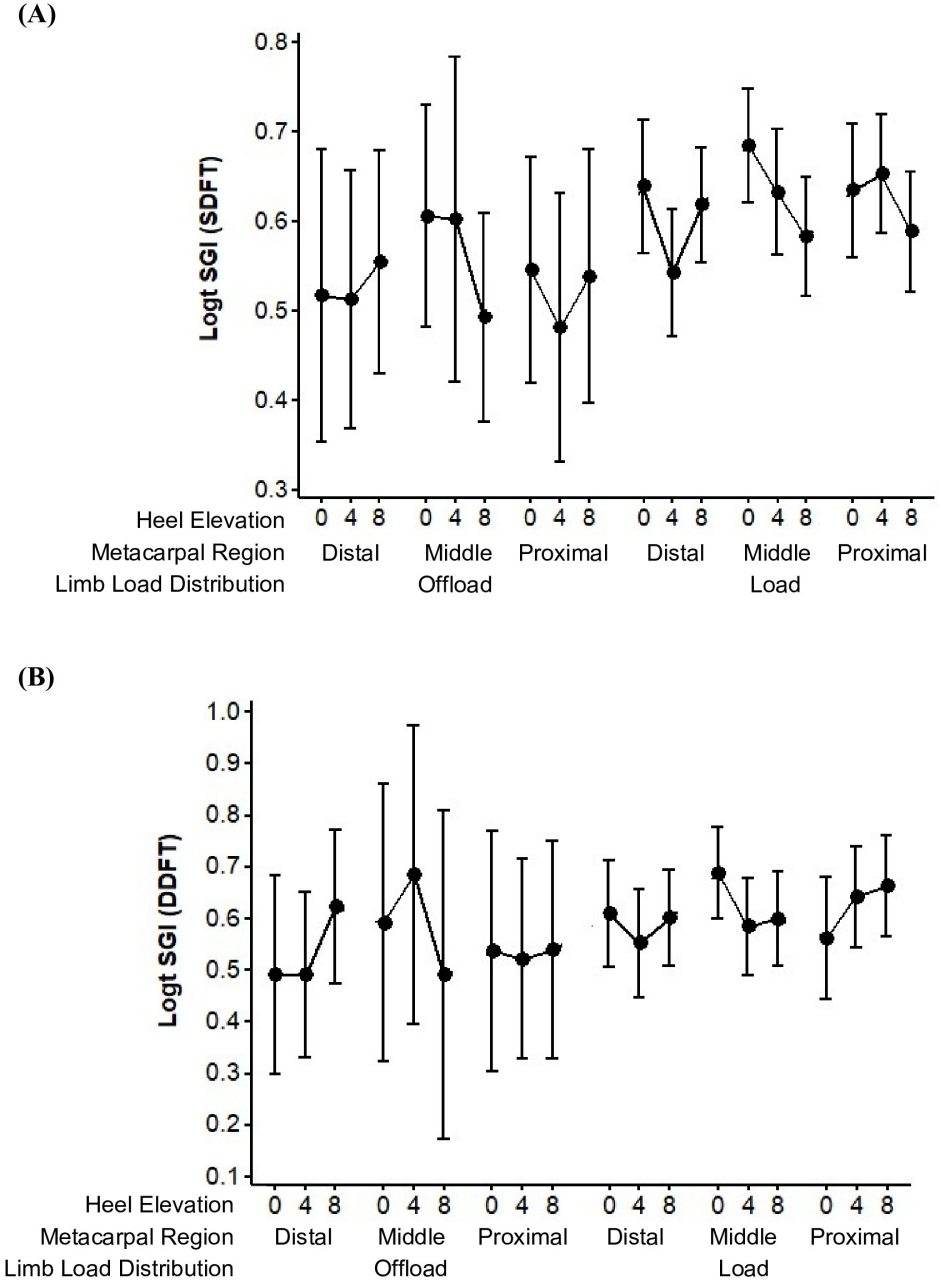
Trends in the mean ± 95% CI of the Strain Gradient Index (SGI) in the Superficial Digital Flexor Tendon (SDFT) **(A)** and Deep Digital Flexor Tendon (DDFT) **(B)**, combined left and right forelimbs.

Effects on SGI for the SDFT and DDFT were evaluated separately, the data for which is depicted in Figure 2. The wide confidence intervals reflected the large variance and the low level of precision among the repeated measures of SGI. There was less variable data observed when the limb was loaded for both SDFT and DDFT, as noted previously.

The mean SGI for the SDFT, within each of the three metacarpal regions, tended to decline when the angle of heel elevation increased from 0 to 8 degrees (Figure 2A). A decrease in mean SGI was observed from 0 to 4 degrees in all circumstances with the exception of the proximal metacarpal region when the evaluated limb was loaded. A decrease in mean SGI was observed from 0 to 8 degrees in all circumstances with the exception the distal metacarpal region when the evaluated limb was off-loaded. More variability in SGI values was observed between 4 to 8 degrees of heel elevation, however, were found to decrease consistently and to the greatest degree in the mid- metacarpal region.

The mean SGI for the DDFT, within each of the three metacarpal regions, sometimes increased and sometimes decreased when the angle of heel elevation increased from 0 to 8 degrees (Figure 2B). A decrease in mean SGI was observed from 0 to 4 degrees in all circumstances with the exception of the middle metacarpal region when the evaluated limb was off-loaded and the proximal metacarpal region when the evaluated limb was loaded (as observed with the SDFT). A decrease in mean SGI was observed from 0 to 8 degrees in all circumstances with the exception the distal metacarpal region when the evaluated limb was off-loaded (as observed with the SDFT) and in the proximal metacarpal region when the evaluated limb was loaded. Overall, the mean SGI tended to decline from 0 to 8 degrees of heel elevation, with the most consistent decrease in SGI occurring between 0 and 4 degrees. The greatest decline in SGI occurred in the mid-metacarpal region.

Radiography data was evaluated, which revealed consistent changes of digital angles with an increase in heel elevation from 0 to 8 degrees (Table 2). The resultant change in digital alignment, in degrees and using a 95% confidence interval for statistical analysis, was a decrease in the dorsal MCP joint angle (−2.301 ± 1.285), an increase in palmar PIP joint angle (0.448 ± 1.016), an increase in palmar DIP joint angle (0.243 ± 2.851), an increase in palmar angle (PA) of the distal phalanx (P3) (6.439 ± 0.768), and an increase in toe angle (TA) (7.691 ± 0.910).

**Table 2:** Change in digital angles from 0 to 8 degrees heel elevation.

Measurement	Mean	Standard deviation	Variance
Dorsal MPJ Angle	−2.301	3.470	12.041
Palmar PIPJ Angle	0.448	2.741	7.517
Palmar DIPJ Angle	0.243	7.695	59.224
Palmar Angle of P3	6.439	2.073	4.297
Toe Angle	7.691	2.457	6.039

## 4 Discussion

Results of the study reported here indicated that both the degree of heel elevation applied and the application of load on the limb had a significant effect on the stiffness gradient of both the SDFT and DDFT in clinically normal horses, derived by AEG evaluation. Despite the wide confidence intervals indicating large variance among the repeated measures of SGI, the mean SGI for both flexor tendons tended to decline with the angle of heel elevation increasing from 0 to 8 degrees. The mean stiffness gradient for both flexor tendons had the most consistent and significant decline in the mid- metacarpal region. Some variability in trends of SGI values was observed for both flexor tendons, with greater deviations observed between 4 to 8 degrees of heel elevation. The relationship between increased heel elevation from 0 to 8 degrees and the SGI values for both SDFT and DDFT, at the level of the mid-metacarpus, is consistent with the hypothesis that increasing heel elevation from 0 to 8 degrees results in an overall decrease in SGI and subsequent tendon strain of both digital flexor tendons in horses. Variations from this trend were observed for both flexor tendons in the distal and proximal metacarpal regions, which was consistent with the hypothesis for the SDFT that both attenuation and amplification of strain would be seen. The hypothesis was nevertheless rejected regarding the DDFT in presuming that only an attenuation in strain would be observed and that a linear relationship would exist between an increase in heel elevation and an attenuation of strain.

While an overall decline in SGI of both flexor tendons was observed from 0 to 8 degrees of heel elevation, the most consistent decrease in SGI occurred between 0 and 4 degrees. A consistent linear correlation does not appear to exist between the stiffness gradient and degree of heel elevation but rather variation exists in the SGI values with an increase in heel elevation, from both 0 to 4 and 4 to 8 degrees, across horses.

Some of the variation in stiffness gradients at differing degrees of heel elevation may be attributable to the range of conformations making up the study population. For example, varying lengths and proportions of the digital phalanges and increased extension of distal joint angles may alter the linear response of the stiffness gradient to changes in heel elevation, and could be an area for future analysis. Radiography data revealed significant variation in the baseline TA and PA of the distal phalanges, which ranged from 38.53 to 61.48 for the TA and 2.1 to 11.3 for the PA in the thoracic limbs evaluated. Additional research would need to be conducted comparing horses of more comparable conformations to better determine the influence of baseline joint angulation on SGI values.

Linear relationships between TA and digital joint angles have been described on numerous accounts (3, 22, 23). Elevation of the heel up to 15 degrees has been shown to cause both a linear decrease in dorsal (or flexion) angle of the MCP joint and a linear increase in palmar angle of the PIP and DIP joints (3, 7, 22, 23). The joint angulation results collected in our study corroborate with these changes in joint angles. When comparing to these studies, a wide variation of the baseline angles of the horses being evaluated similarly exists.

Crevier-Denoix evaluated the effects of heel elevations upon the digital joint angles of horses with different initial toe angles and determined that while the horse effect was significant for all joint angles, there was no significant difference in angular variation compared to baseline. It was therefore concluded by Crevier-Denoix et al. that the response of horses to manipulation of heel position is basically the same, and the therapeutic effect by altering angulation will be similar for all individuals and is not dependent on individual hoof conformation (3). While the joint angulation followed a similar pattern in our study, the effect of heel elevation on the stiffness gradient of digital flexor tendons was found to be more variable and does not have a similar linear relationship. Considering the wide range of conformations in the study population and the variability observed, it is theorized that stiffness gradients and tendon strain of the DDFT and SDFT are affected by and dependent on individual distal limb conformation. More comprehensive analysis comparing digital angle measurements in relation to tendon strain data acquired from AEG will be conducted in a subsequent study.

Increased variability of SGI measures with incremental wedge application at varying metacarpal regions, primarily at the proximal and distal metacarpus, may be influenced by proximity to joints and/or proximity to origin and insertion sites of the digital flexor tendons. As mentioned previously, tendon strain will likely be influenced by conformational differences which may be a larger factor for SGI variability at proximal and distal sites being closer to joints with differing joint positions. Future analysis should examine the interrelationship between statically loaded joint angles and SGI at various metacarpal regions, recognizing that some locations (i.e., proximal and distal) appear to be subject to increased variability in SGI with increasing heel elevation from 0 to 8 degrees.

Some of the variability in stiffness gradient results identified in the present study may be due to a difference in tendon structure and elastic potential at different locations of the metacarpus. As previously discussed, the mean stiffness gradient had a more consistent decline in the mid-metacarpal region and had a less consistent trend at the level of the distal metacarpus. The digital flexor tendons distally, between 18 and 25 cm distal to the accessory carpal bone and adjacent to the sesamoidodigital region, has not been evaluated in previous AEG studies performed in horses (15). The sesamoidodigital portion of the equine SDFT has been shown to have a decreased elastic modulus and stiffness compared to the metacarpal region (15, 24). The stiffness gradient in this region was consistently lower compared to the mid-metacarpal region with a tendency to increase with initial heel elevation from 0 to 4 degrees and then decrease with added elevation from 4 to 8 degrees, which did not support our *a priori* hypothesis.

Bearing in mind the limited studies on the use of AEG in horses, it is apparent that further refinement on data collection and processing may be advantageous to maximize the use of this technique in horses. During acquisition of data, cineloops were recorded while the evaluated limb was placed in both a loaded and off-loaded state. Tendon length increases with the load applied and decreases with the load removed. Results in the present study revealed a narrower CI and therefore less variability when the digital flexor tendons being examined were placed under load. Advances in AEG software have allowed for stiffness gradients to be evaluated with digital flexor tendons either loaded or off-loaded, as a change in length in the tendon is observed under both circumstances. Previous studies using AEG have only examined stiffness gradients with the limb under load, and also had comparatively higher repeatability of stiffness gradient values reported (15, 16). The present study demonstrates that while stiffness gradients can be collected following either application or removal of load, that the AEG data is more accurate and has less variability with the former.

In the present study, all horses included were presumed to have normal digital flexor tendons based on history, findings on physical and orthopedic evaluation, and sonographic assessment. Nonetheless, the application of therapeutic orthotics utilizing heel elevation of 8 degrees and above is typically reserved for cases with a specific musculoskeletal condition or pathology. Additional research is needed to evaluate changes in stiffness gradients with AEG in injured and healing equine tendons, and to better determine how heel elevation affects the SDFT and DDFT in an altered or diseased state.

Expanding this research to evaluate effects of heel elevation greater than 8 degrees on stiffness gradients of digital flexor tendons should also be examined. Pearce et al. has demonstrated that a significant change in joint angulation and subsequent change in tendon strain may only result from application of heel elevation greater than 15 degrees (7). Considering there are equine orthotics available commercially and that are used clinically that increase heel elevation by 20 degrees (Nanric, Ultimate shoe), understanding in greater detail the effect that application of heel elevations measuring between 8 and 20 degrees would have on the SGI of digital flexor tendons would be beneficial.

There are complex reciprocal interactions that occur between the hoof, the phalangeal joints, and the flexor tendons. It has been shown that digital joint angles change significantly in the standing horse following heel and toe elevation, and suggested that those changes alter tension in the surrounding flexor tendons (3). Data derived from kinematic modeling of the distal limb has demonstrated that the application of a heel wedge decreases strain in the DDFT, yet consequently increases strain in the SDFT (3, 21, 25). These results are inconsistent with other studies, such as those using strain gages to calculate tendon strain. Riemersma et al. implanted mercury-in-silastic strain gages in the digital flexor tendons and suspensory ligament (SL) to examine tendon strain following shoeing modifications, including the application of a 7-degree heel wedge. A decrease in strain was found in the DDFT and an increase in strain found in the SL, but no significant change was identified in the SDFT with the wedge (12). Thompson et al. utilized Hall-effect strain sensors, and similarly found a decrease in DDFT strain with heel elevation but no significant change in SDFT strain (11).

Hagen et al. examined the effect of heel wedges on the cross-sectional area of the digital flexor tendons, and found that increasing heel elevation up to 20 degrees resulted in an increased cross-sectional area for the SDFT and DDFT in the mid-metacarpal region. These results indicate relaxation and a presumed decrease in strain of both digital flexor tendons. The results presented in our study support the theory proposed by Hagen et al., that an increase in heel elevation causes a decrease in tendon strain for both digital flexor tendons in the mid-metacarpal region (26).

While there remains a lot of inconsistencies regarding the observed effect of heel elevation on SDFT strain, it is clear that distinct anatomical differences between the digital flexor tendons exist. A difference in response to heel elevation for each flexor tendon is therefore expected taking into consideration the variation in tendon attachments, structure and composition. The DDFT has been shown to have a lower elastic modulus and higher stiffness gradient compared to the SDFT (27, 28). The results from the present study, evaluating SGI for the loaded SDFT and DDFT, reveal similar trends in decreasing stiffness gradient with heel elevation but also show differing ranges of SGI values for each tendon.

When a veterinarian deliberates making an alteration in hoof conformation, as with application of a therapeutic orthotic for treatment or prevention of a musculoskeletal problem, it is imperative they consider the effects on the specific anatomical site of interest while also recognizing the subsequent effects that those changes may have on other structures. Bushe et al. has proposed that that biomechanical rationale for a change in digital alignment with heel elevation was due to decreased tension on the proximal suspensory ligament, allowing dorsal translation of the distal aspect of the proximal phalanx (21). Pearce et al. suggested that with greater levels of heel elevation that the palmar soft tissues will change from being under tension to the dorsal soft tissues being under tension (7). While the stiffness gradient of DDFT and SDFT were both evaluated in this study, additional soft tissue structures (i.e., suspensory ligament) were not examined. Additional research will be required to further understand the preponderant clinical effects that decreasing strain on the digital flexor tendons by application of a heel wedge will have on other structures in both acute and chronic state. The authors maintain that a quantitative understanding of the interrelationship between the changes in palmar heel angle, phalangeal joint angles, digital flexor tendon strain, and remaining surrounding soft tissue structures in live, standing horses, would be valuable for clinical decision making.

The present study had several limitations. One of the limitations was the small sample size, which reduced the likelihood of finding any effect or influence of patient factors on the AEG findings and the effects of such differences need to be further explored. Differences in age, sex, breed and exercise regimens are all factors that may influence the stiffness gradients measured by use of AEG. While AEG is ideal in providing a non-invasive means of evaluating digital flexor tendon strain, the tendons are only evaluated in a static state. Lochner et al. implanted high-elongation strain gages in the SDFT, DDFT, and SL to evaluate the effects of changing hoof angle on strain, and then examined the horses in motion to evaluate the relationship between tendon strain on the phases of the stride (29). Variations in tendon strain were observed at different phases of the stride, which is presumed but cannot be tested with the AEG technique.

It should also be recognized that repeated use of sedation was required for AEG data acquisition in this study. Degasperi et al. reported that with the single sedation regimen employed that a consistent influence on the stiffness gradient of the SDFT was observed. Images obtained after sedation in that study was acquired within 30 min after drug administration (16). Collection of data in the present study occurred over longer time periods and required multiple boluses of sedation. Whether administration of multiple doses would change SGI values or contribute to greater variations in stiffness gradient data corresponding with the peaks and troughs of plasma levels of sedative drugs in the blood is unknown. While the initial sedation protocol modeled that of the Degasperi study, the duration of sedation required to acquire AEG data for horses at varying levels of heel elevation was considerably longer, and for most subjects data acquisition occurred over a span of multiple days.

Another limitation was the variability of patient compliance during collection of AEG data. A number of subjects resisted loading and offloading of the examined limb, and therefore the maximal stretch targeted for the flexor tendons may have not been acquired for all horses which may have an effect on stiffness gradients. Horses included in the present study had physiologically normal digital flexor tendons as determined based on history, results of an orthopedic evaluation, and results of a complete ultrasonographic examination. However, histologic evaluation was not performed. Despite the exclusion of horses with obvious abnormalities, subclinical tendon injury, while deemed unlikely, could have been present in some of the horses evaluated.

The AEG technique can be easily and effectively utilized to measure digital flexor tendon strain in a standing horse with applied shoes and orthotics (i.e., heel elevation), and can be related directly to joint angles through simultaneous radiographic evaluation. In being a non-invasive approach, more extensive research opportunities exist for evaluating larger populations of horses and at greater variations of heel elevation. This model resulted in direct quantification of tendon strain as it relates to hoof conformation, which consequently allows for more targeted therapeutic farriery techniques.

## Data Availability

The raw data supporting the conclusions of this article will be made available by the authors, without undue reservation.
